# Semen characteristics and sperm morphological studies of the West African Dwarf Buck treated with Aloe vera gel extract

**Published:** 2011

**Authors:** Oyeyemi Matthew Olugbenga, Samuel Gbadebo Olukole, Ajayi Tolulope Adeoye, Adeniji Deborah Adejoke

**Affiliations:** 1 Department of Veterinary Surgery and Reproduction, University of Ibadan, Nigeria.; 2 Department of Veterinary Anatomy, University of Ibadan, Nigeria.

**Keywords:** *Aloevera*, *Spermmorphology*, *Bucks*

## Abstract

**Background: **Aloe vera (*Aloe barbadensis miller*) is an evergreen perennial plant widely used in modern herbal practice and is often available in proprietary herbal preparations.

**Objective**: This study was designed to investigate the semen picture and spermatozoa morphology of West African Dwarf (WAD) bucks treated with Aloe vera extract.

**Materials and Methods**: Twelve sexually matured WAD bucks, weighing between 11 and 15 kg, were used for the study. The bucks were first used as control (pre-treatment) and later as two groups of six animals each. The first six bucks received 10 mls of the 3% extract while the other six received 10 mls of the 4% of the extract for a 7 day period. Semen was collected from both the 3 and 4% extract treated bucks for the control (pre-treatment), on days eight (first week post-treatment) and fifteen (second week post-treatment) in each case using the electroejaculation method. The spermiogram of the bucks were investigated using standard procedures. Data obtained were analyzed using two way ANOVA and significance reported at p<0.05.

**Results**: The continuous administration of Aloe vera extract significantly (p<0.05) reduced sperm concentration, motility and percentage livability and resulted in increased sperm abnormalities in the WAD buck.

**Conclusion**: Aloe vera adversely affected the spermiogram of bucks. The plant can reduce fertility in male animals and is therefore not recommended for medicinal purpose in male animals especially those used for breeding.

## Introduction

Aloe vera (*Aloe barbadensis miller*) is an evergreen perennial plant, growing to 0.8m to 1m at a slow rate. It is a fairly well known herbal preparation with a long history of use. Aloe vera is widely used in modern herbal practice and is often available in proprietary herbal preparations (1). The clear gel contained within the leaf makes an excellent treatment for wounds, burns and other skin disorders, placing a protective coat over the affected area, speeding up the rate of healing and reducing the risk of infection (2). Apart from its external use on the skin, Aloe vera (usually the bitter aloes) is also taken internally in the treatment of chronic constipation, poor appetite, and digestive problems (3). 

 The West African Dwarf (WAD) goats occurring in the tropical forest belt of West Africa are small sized breeds with 10-30kg weight. The WAD goat is very important in developing countries, being able to thrive in adverse conditions and has a high fertility rate with a short generative interval allowing for a possible increase in population compare with cattle in West Africa (4). The sperm cell can be broadly divided into the head, and tail with the tail further divisible into 4 regions: the neck, the middle piece, the principal piece and end piece (5). The head is covered by a protoplasmic cap known as the Galea capitis having shapes varying according to species. It is ovoid in the bull, ram, rabbit while in man it is round. The middle piece of the spermatozoa provides energy for motility while its distal end piece consists of inner axial filaments resembling the flagellum of a ciliated cell (6).

Sperm morphological studies had been reported in WAD bucks treated with pumpkin plant (*Cucurbita pepo*) and in West African Dwarf rams treated with *Euphorhia hirta *(4, 7). Nevertheless, there is a dearth of information on the effect of the Aloe vera plant on male fertility especially on semen picture and spermatozoa morphology. This study, therefore, was designed to investigate the semen picture and spermatozoa morphology of WAD bucks treated with Aloe vera extract.

## Materials and methods

**Animal protocol**

Twelve sexually matured WAD bucks, weighing between 11 and 15 kg, were used for the study. They were kept at the Large Animals Ward II of the Veterinary Teaching Hospital (VTH), University of Ibadan, located between latitude 150N and 300S with relative humidity ranging from 50-80%, rainfall is about 70 inches per annum and temperature between 28°C and 34°C. The animals were kept on guinea corn offal and grasses and water was provided *ad libitum*. The animals were dewormed using Albendazole and Levamisole while Ivomec was used to control ectoparasites and mange. They were vaccinated against pests petit ruminants (PPR) using PPR vaccine (NVRI, Vom, Nigeria) among other veterinary attentions.

**Preparation and administration of Aloe vera extract**

The extract was collected from of Aloe vera plant by cutting the leaves open and the inner part scrapped. 3.0g and 4.0g of the scrapped Aloe vera gel were mixed with 100ml of distilled water to give 3% and 4% solutions of the extract respectively. 

The bucks were first used as control (pre-treatment) and later as two groups of six animals each. The first six bucks received 10 mls of the 3% extract while the other six received 10 mls of the 4% of the extract. The treatment period lasted 7 days.

**Semen collection**

Semen was collected from both the 3 and 4% extract treated bucks for the control (pre-treatment), on days eight (first week post-treatment) and fifteen (second week post-treatment) in each case using the electroejaculation method as described by Zemjanis (9). 

**Morphological studies**

On a clean, warm glass slide, a drop of semen was placed as well as two drops of Wells and Awa stain as reported by Hammer (6). The semen and stain were thoroughly mixed together with a smear made on another clean and warm slide. The smear was air-dried and observed using the light microscope starting with low power to high magnification. The presence of abnormal cells out of at least 400 sperm cells from several fields on the slide was counted and their total percentage was estimated.

**Motility**

Sperm motility was assessed by the method described by Zemjanis (9) and was evaluated microscopically within 2-4 minutes of their isolation from the cauda epididymis and later expressed as percentages.

**Sperm count**

Epididymal sperm count was obtained by mincing the cauda epididymis in distilled water and filtering through a nylon mesh. The spermatozoa were counted by haemocytometer using improved Neubauer chamber (Deep 1/10mm, LABART, Germany) described by Pant and Srivastava (10).

**Sperm live/dead ratio**

A drop of semen was placed in 1% eosin and 5% nigrosin in 3% sodium citrate dehydrates solution for the live/dead ratio as described by Wells and Awa (11).

**Statistical analysis**

All data obtained were expressed as means with the standard errors and were subjected to analysis of variance (ANOVA) according to the standard procedure described by Steel and Torrie (12). Duncan multiple range test was used to compare means found to be statistically significant (p< 0.05) as described by Obi (13). 

## Results

The mean volumes of ejaculate observed throughout the study were 0.3±0.02ml and 0.23± 0.02ml and 0.15±0.02ml for the pre-treatment, first week post treatment and second week post treatment with Aloe vera extract respectively (Table I). The color of semen observed throughout the three stages of the study varied from a homogenous milky to creamy white fluid. The mean percentage progressive sperm motility observed in this study were 85, 80 and 70 for the pre-reatment, first week post treatment and second week post treatment with Aloe vera extract respectively. These values showed significant differences (p<0.05) across the stages of the work. The mean percentage spermatozoa livability also decreased from 96 to 90 and then 83 for the pre–treatment, first week post treatment and second week post treatment with Aloe vera extract respectively. The average sperm concentrations were 428±4.26×10^9^ spermatozoa/ ml, 341±6.63 ×10^9^ spermatozoa/ml and 289±4.81×10^9^ spermatozoa/ ml; for the pre–treatment, first week post-treatment and second week post treatment with Aloe vera extract respectively. The sperm morphological characteristics observed in this study for the 3 and 4% extract treated bucks are given in tables 2 and 3 respectively. 

**Table I:** Mean and SEM values for semen characteristics in Aloe vera extract treated WAD bucks

**Parameters**	**Pre-treatment**	**First week** **post-treatment**	**Second week** **post-treatment**
Volume of ejaculate (ml)	0.30 ± 0.02 *	0.23 ± 0.02	0.15 ± 0.02*
Color of ejaculate	Milky to creamy white	Milky to creamy white	Milky to creamy white
Sperm motility	85% *	80%	70% *
Spermatozoa viability	96% *	90%	83% *
Sperm concentration (x 10^9^/ml)	428 ± 4.26 *	341 ± 6.63 *	289 ± 4.81*

* Significantly different (p<0.05).

**Table II:** Sperm morphology in 3% Aloe vera extract treated WAD bucks.

**Parameters**	**Pre-treatment**	**First week** **post-treatment**	**Second week** **post-treatment**
Tailless head	6.00±1.00^a^	8.33±1.86^a^	10.33±0.88^a^
Headless tail	5.00±0.58^a^	5.33±0.67^b^	9.00±1.53^b^
Rudimentary tail	2.33±0.67^a^	2.33±0.67^b^	2.67±0.33^c^
Bent tail	8.33±0.33^a^	10.67±0.67^a^	10.00±1.00^b^
Curved tail	7.33±0.67^a^	10.67±1.20^a^	10.67±1.20^b^
Curved mid-piece	7.33±0.33^a^	11.33±0.33^a^	13.00±0.58^a^
Bent mid-piece	7.33±0.88^a^	10.33±1.33^a^	10.67±0.67^b^
Coiled tail	1.00± 0.33^a^	1.33±0.33^b^	1.67±0.33^c^
Total abnormal cells	44.00±2.31^a^	60.33±3.84^a^	68.00±4.16^a^
Total normal cells	756.00±2.31^a^	739.67±3.84^b^	732.00±4.16^c^
Total cells	800.00±0.00	800.00±0.00	800.00±0.00

Means with the same superscript within the rows are statistically significant (p<0.05).

**Table III:** Sperm morphology in 4% Aloe vera extract treated WAD bucks

**Parameter**	**Pre-treatment**	**First week** **post-treatment**	**Second week** **post-treatment**
Tailless head	6.33±0.67^a^	5.33±0.88^a^	7.33±1.20^a^
Headless tail	5.33±0.33^a^	5.33±0.33^b^	8.33±1.33^c^
Rudimentary tail	2.33±0.33^a^	2.67±0.33^b^	3.00±0.00^c^
Bent tail	8.33±0.88^a^	10.33±0.88^a^	12.00±1.53^a^
Curved tail	6.33±0.33^a^	10.67±0.33^a^	10.33±1.20^b^
Curved mid-piece	8.00±0.58^a^	12.67±1.67^a^	13.00±1.00^b^
Bent mid-piece	6.67±1.20^a^	12.00±1.53^a^	12.00±1.33^b^
Coiled tail	2.00±1.00^a^	2.00±0.58^b^	2.33±0.33^c^
Total abnormal cells	44.67±2.19^a^	61.00±3.06^a^	68.33±4.10^a^
Total normal cells	755.33±2.19^a^	739.00±3.06^b^	731.67±4.10^c^
Total cells	800.00±0.00	800.00±0.00	800.00±0.00

Means with the same superscript within the rows are statistically significant (p<0.05).

## Discussion

The semen characteristics of WAD bucks in this study were similar to those previously reported in WAD bucks by previous authors (4, 7, 17). The color of the semen observed at the end of first week and second week of dosing the WAD bucks with Aloe vera extract was not different from the pre-treatment semen color. This agrees with the findings of Arthur (13) on the color and consistency of semen in bucks. Therefore it can be suggested that the continued administration of Aloe vera extract does not have any effect on the colour of semen of WAD bucks. This is similar to the report of Oyeyemi *et al* (7) on the effect of the pumpkin plant (Cucurbita pepo) extract on the semen of WAD bucks. However, there were significant differences (p<0.05) in the volume of the semen observed from the pre-treatment to the second week post-treatment with Aloe vera extract. This can however be linked to the delayed ejaculation time compared to the ejaculation time before the onset of the administration of the extract. 

The mean percentage progressive sperm motility observed during the pre-treatment and first week post–treatment period of this study were within the normal range for WAD bucks. However, the value significantly reduced in the WAD bucks during the second week post-treatment with Aloe vera extract. This suggests that treatment with Aloe vera adversely affect sperm motility. Moreover, sperm capacitation, the series of enzymatic reactions resulting in the release of acrosomal enzymes which allow for fertilization in the female reproductive tract will be adversely affected. The delay in the occurrence of capacitation had been reported to render spermatozoa nonfunctional (14). The mean percentage sperm livability of the bucks used in the study had a significant (p<0.05) decrease during the second week post-treatment compared to same values during pre-treatment and the first week post treatment with Aloe vera extract. The mean concentration of sperm showed significant differences (p<0.05) across the stages of the work. This indicates that the continuous administration of Aloe vera extract would result in decreased fertility in the WAD buck. 

The presence of abnormal forms of spermatozoa in the pre-treatment stage of this work is consistent with the report of Moss *et al* (15) that a number of abnormal forms of spermatozoa are normally encountered in all ejaculates. When these abnormal spermatozoa are present in large numbers, they are associated with impaired fertility. The 3% Aloe vera extract treated bucks had mainly secondary sperm abnormalities of headless tail and curved mid-piece. There was also a significant (p<0.05) increase in total abnormal spermatozoa. Significant differences were observed (p<0.05) in the mean values of headless tail and curved mid-piece spermatozoa during pre-treatment and first week post-treatment and second week post-treatment (Table I). The 4% Aloe vera extract treated bucks also had mainly secondary sperm abnormalities of curved tail, curved mid–piece and bent mid-piece. Significant differences were observed (p<0.05) in the mean values of curved tail, curved mid-piece and bent mid-piece spermatozoa during pre-treatment and first week post-treatment and second week post-treatment (Table II). There was also a significant (p<0.05) increase in total abnormal spermatozoa on the administration of the 4% Aloe vera extract. 

High numbers of secondary abnormalities have been reported to adversely affect spermatozoa storage in the epididymis (14). There is no convincing evidence for significant absorption of spermatozoa, by spermiophagy or dissolution in the epididymis of normal animals. Spermiophagy by epithelial cells or intraluminal macrophages may take place if the duct ruptures and granulomas form (15). The epididymis stores sperm, primarily in the cauda region. In most mammals, including farm animals, viable spermatozoa can be retained in the epididymis for 2 to 3 weeks (16). However, in smaller rodents, if epididymal sperm is trapped in the epididymis (by ligation of the vas deferens) for up to 50 days, it will still express motility when finally released from the cauda epididymidis (17). The cauda epididymidis not only stores the sperm but also provides the proper conditions for holding sperm in a quiescent state of metabolism. However the duration that the epididymis can maintain viable sperm is variable between species (14). Occurrence of sufficiently high number of mid-piece spermatozoa abnormality has been traced to the period of storage in the epididymis (18). Mid- piece abnormalities had also been traced to the deficiency of zinc. Zinc and folate are involved in the synthesis of DNA and RNA. Although the extract pathophysiology of zinc deficiency leading to clinical symptoms of decreased spermatogenesis and impaired male fertility has not been known but it has been shown to cause impaired male fertility in the form of reduced sperm motility, reduced percentage motility of sperm, morphological abnormalities and reduced spermatogenesis (19). The process of spermatogenesis in a buck takes 49 to 63 days, while spermatozoa are formed daily during the life of bucks (7).

It can be concluded that the continuous administration of Aloe vera extract can induce oligospermia in animals having significantly (p<0.05) reduced sperm motility, percentage livability of sperms and sperm concentration. The plant can reduce fertility in male animals and is therefore not recommended for medicinal purpose in male animals especially those used for breeding.
